# Penicillin Binding Protein from *Pediococcus acidilactici* Isolated from Nuruk for Food Biopreservative

**Published:** 2018-11

**Authors:** Da Hye SONG, Jeung Min LEE, Kang Hyun CHUNG, Jeung Hee AN

**Affiliations:** 1. Dept. of Food Science and Technology, Seoul National University of Science & Technology, Seoul, Republic of Korea; 2. Division of Food Bioscience, Konkuk University, Chungju, Republic of Korea

**Keywords:** Nuruk, *Pediococcus acidilactici*, Bacteriocin, Penicillin binding protein

## Abstract

**Background:**

Korean traditional nuruk, consisting of a variety of microorganisms, is widely used in traditional liquor materials. The present study evaluated the antimicrobial activity of strains isolated from Korean traditional nuruk in 2016.

**Methods:**

The strain was isolated from Korea traditional nuruk and performed antimicrobial activities using the paper disc test and phylogenetic analysis using 16S rRNA sequencing. The bacteriocin was identified by matrix-assisted laser desorption/ionization time-of-flight mass spectrometry.

**Results:**

The isolate, S-2, demonstrated highest antibacterial activity against various gram-positive and gram-negative pathogens, including *Klebsiella pneumoniae, Salmonella enterica subsp. enterica, Bacillus subtilis, B. cereus, Escherichia coli and Shigella flexneri.* The isolated was identified as *P. acidilactici*, by 16S rRNA sequence analysis. Antibacterial activity of *P. acidilactici* was retained over a wide temperature range. And the *P. acidilactici* strains remained active over a wide pH range. However, reduced activities were obtained at alkaline pH. When the bacteriocins from this strain were treated with proteolytic enzymes, loss of antibacterial activity was observed. No effect in the activity, however, was observed upon treatment with α-amylase, β-amylase, lipases, proteases, and proteinase K. The molecular weight of bacteriocins was estimated to be approximately 51 kDa. Using MALDITOF/MS, the bacteriocins were identified as a putative penicillin binding protein.

**Conclusion:**

This study is the first report of isolation of bacteriocin with the above mode of actions from Korean traditional nuruk. The bacteriocins produced by the strain have potential applications in food preservation.

## Introduction

*Pediococcus* is a genus of gram-positive lactic acid bacteria, belonging to the family Lactobacillaceae. The genus comprises of eight species, namely *P. acidilactici, P. pentosaceus, P. damnosus, P. parculus, P. inopinatus, P. halophilus, P. detrinicus* and *P. urinaeequi.* Among these species, *P. acidilactici* is involved in food fermentation (1). *P. acidilactici*, isolated from milk, meat products, lamb, and beer, has a strong tolerance towards acidic and bile salt conditions (2). Pediocin AcH, produced by *P. acidilactici*, was characterized to belong to class IIa bacteriocins, due to its broad range antibacterial activity, stability, and potential for use as a food bio-preservative (3). In addition, *P. acidilactici* strain MA18/5M was characterized as a probiotic with significant beneficial effects on gut health and microbiota, resulting in improved growth performance of broiler chickens (4). Similarly, in vivo studies involving dietary supplementation of *P. acidilactici* demonstrated improved egg weight, feed efficiency, yolk color, egg shell quality, and decreased number of damaged eggs and yolk cholesterol levels in laying hens (4). Besides, the strain has been demonstrated to promote shrimp growth rate and yield (5).

Bacteriocins comprise a huge family of ribosomally synthesized peptides, with antimicrobial activity against other bacteria found in nature (6,7). Bacteriocins, produced by lactobacilli, are mainly sub-divided into 4 classes. Class I bacteriocins also referred to as “lantibiotics”, were isolated from the members of Lactobacillaceae family (8). Another class, Class II bacteriocins or the non-lantibiotics, belong to the heat-stable and unmodified class of bacteriocins (1). Class IIa bacteriocins are produced by food-associated strains and are isolated from a variety of food products from industrial and natural origins, including vegetables, meat, and dairy products (8). Class III bacteriocins are high molecular weight, heat-labile proteins found in lactobacilli. Class IV bacteriocin is a complex group of proteins related to other lipid or carbohydrate moieties (9). Bacteriocins produced by *Pediococcus* sp. are heat-stable, small, non-lanthionine peptides belonging to the class II bacteriocins. These biologically active peptides demonstrate a bactericidal mode of action against common food-borne pathogens, such as *B. cereus, C. perfringens, Listeria species*, and *S. aureus* (10–15).

The aim of the present study was to identify and characterize pediococci from Korean traditional nuruk, followed by determination of bacteriocin production from the isolates and assessment of the antimicrobial activity against gram-positive and gram-negative bacteria. The bacteriocins produced by *Pediococcus* strains were purified and characterized. This study reports, for the first time, the isolation of a penicillin binding protein inhibiting bacteriocin from Korean traditional nuruk.

## Materials and Methods

### Isolation of Pediococcus strains

Korean traditional nuruk was provided at Iksan, Jeollabuk do (2017). The sample was crushed, collected in sterile cap tubes, and dissolved in sterile water. The diluted samples were plated onto de Man, Rogosa and Sharpe (MRS) agar plates, followed by incubation at 37 °C for 24 h. The colonies obtained were cultured in MRS broth and incubated at 37 °C for 24 h.

### DNA extraction and 16S rRNA Polymerase chain reaction (PCR)

The 16S rRNA coding region was amplified using PCR (Thermal Cycler, Takara Shuzo Co., Ltd., Japan) (16). The PCR products were sequenced directly with a sequence kit (AL Fexpress Auto Cycle; Pharmacia Biotech, Piscataway, N.J.) using the prokaryotic 16S ribosomal DNA universal primers 27F (5′-AGAGTTTGATCCTGGCTCAG-3′) and 1492R (5′-GGTTA CCTTGTTACGACTT-3′). Nucleotide substitution rates (Knuc values) were calculated, and the phylogenetic tree was constructed by neighbor-joining method. Topologies of the trees were evaluated by bootstrap analysis using CLUSTAL W software+, based on 100 random re-samplings. The sequences were aligned with the published sequences from DDBJ, GenBank, and EMBL.

### Effect of temperature, pH, and enzyme treatment on bacteriocin activity

Bacteriocins identified in the present study were evaluated for their thermal and pH stabilities and susceptibility to denaturation by enzymes. The cells were harvested by centrifugation at 3500 rpm for 15 min, followed by aliquoting 1 ml of the cell-free culture supernatant. This was followed by incubating the supernatant at varying temperatures (−65 °C, 0 °C, 25 °C, 36 °C, and 100 °C) to monitor the temperature stability of the bacteriocins. The effects of pH on the activity of bacteriocins were tested by adjusting the pH of the cell-free supernatants from pH 4.0 to 10.0 using sterile 1 M NaOH or 1 M HCl. Afterward, the pH of the samples was re-adjusted to 7.0, and antimicrobial activity was tested by disc diffusion method.

The cell-free supernatants of the isolated *Pediococcus* strains were treated with different enzymes, such as trypsin (Welgene, Korea), α- amylase (Junsei, Japan), β-amylase (Sigma-Aldrich, USA), proteinase K (Gendepot, USA), protease (MP bio, USA), papain (Wako, Japan), and lipase (Sigma) – 400 units. This was followed by assessment of the antibacterial activities of the samples using disc diffusion method.

### Purification of bacteriocins from P. acidilactici

The strains were grown in MRS medium and harvested at 6500 rpm for 20–30 min. The cell-free supernatant was precipitated using C18 column. The column was washed by methanol and then rinsed with distilled water, followed by media solution. Elution was performed using acetonitrile. The fractions collected post-elution were concentrated using a vacuum concentrator (Ecopspin 314, PS1E1AF01, Biotron Inc., Korea). The samples were separated on a 12% SDS-PAGE gel. Protein marker (Gendepot, USA) with a size range of 250 kDa was used. The gel was stained with Coomassie Brilliant Blue G-250. The selected gel was placed on plate count agar with *E.coli* as the indicator strain*.* The plate was incubated at 37 °C for 24 h and the ZOI were examined.

### Matrix-assisted laser desorption/ionization time-of-flight mass spectrometry (MALDITOF MS)

For MALDI-TOF MS, in-gel digestion of sample was performed. Tryptic peptides were analyzed in an ultrafl Xtreme^™^ MALDI TOF/TOF device (Bruker, Bremen, Germany) using Flex Control software v3.3 (Bruker). Protein identification was performed using Mascot search engine (Matrix Science, London, UK), using the following parameters: monoisotopic mass accuracy, fragment mass tolerance ± 0.5 Da; missed cleavages 0, allowed variable modifications, oxidation (Met), and carbamidomethylation (Cys). Positive identification was retained with MASCOT scores above the threshold level (*P*<0.05).

### Statistics analysis

All values are analyzed using SPSS (ver. 18.0, Chicago, IL, USA). The means and standard deviations were calculated and the significance of each zone was verified using Duncan test method at the 5% level of significance (*P*<0.05).

## Results

### Identification of Pediococcus strains

**Fig. 1:**
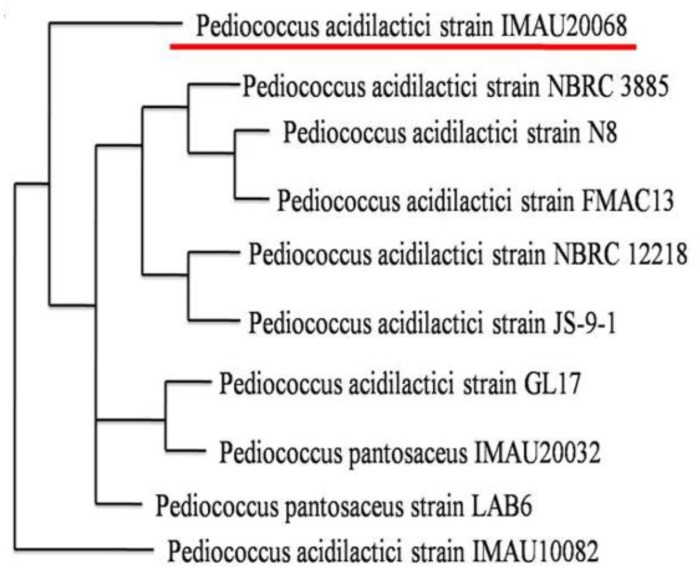
Phylogenetic tree of S-2 based on *Pediococcus* strains / S-2 was identified as *P. acidilactici*

Ten strains, isolated from Korean traditional nuruk, were screened for their antibacterial activities by the disc diffusion method (data not shown). The strains S-2 exhibited the highest antibacterial activity against different gram-positive and gram-negative pathogens, *K. pneumonia, S. enterica subsp. enterica, B. subtilis, B. cereus, E. coli*, and *S. flexneri*. Since these isolates exhibited similar antagonist activities against the tested indicator pathogens, they were selected for further analysis. The nucleotide sequences of the PCR products were compared with other 16S rRNA sequences retrieved from GenBank database. The phylogenetic tree (Fig. 1) was constructed from evolutionary distance analysis by neighbor-joining method.

Following phylogenetic analysis, the representative strain, S-2, was placed in the cluster of the genus *Pediococcus*. Sequence analysis of the strain S-2 using BLAST analysis with reference strains deposited in the GenBank revealed 100% homology of the isolate with P. *acidilactici*.

### Effects of temperature, pH and enzymes treatments of Pediococcus.

The activity of bacteriocins from *P. acidilactici* was tested at different temperatures, pH, and enzyme treatments (Table 1). Cell-free supernatant obtained from *P. acidilactici* was tested for temperature stability. *P. acidilactici* retained its antibacterial activity at a wide temperature range of −65 °C −100 °C (Table 1). Analysis of the effect of pH on stability of bacteriocins was performed in the pH range 4–10. *Pediococcus* strains remained active over a wide range of pH conditions. However, reduction in activity was observed at alkaline pH. *P. acidilactici* showed excellent stability. Therefore, pH modulation affects the activity of bacteriocins (Table 1).

**Table 1:** Effect of temperature, pH and enzyme treatments on antimicrobial activity of bacteriocins produced by *P. acidilactici*

***Variable***		***P. acidilactici (%)***
*Temperature*	*Control*	*100^a^*
	−65 °C	93.5^ab^
	0 °C	86.9^b^
	25 °C	86.9^b^
	36 °C	100^a^
	60 °C	92.8^ab^
	100 °C	88.2^b^
pH	Control	100^a^
	4.0	85.0^b^
	5.0	89.5^ab^
	6.0	78.4^c^
	7.0	73.0^c^
	8.0	76.3^c^
	9.0	69.7^d^
	10.0	71.9^cd^
Enzyme	Control	100^a^
	α-amylase	98.0^a^
	β-amylase	89.3^a^
	Papain	0^d^
	Protease	82.8^b^
	Protease K	87.1^b^
	Lipase	78.4^c^
	Trypsin	0^d^

Different superscripts in a column indicate significant differences (*P*<0.05)

In order to confirm the proteinaceous nature of the antimicrobial component isolated from *Pediococcus* strains, the cell-free supernatant was treated with different proteolytic enzymes. A loss of antibacterial activity was recorded when the supernatant was subjected to the treatment with papain and trypsin. The activity, however, was retained upon treatment with α-amylase, β-amylase, lipase, protease, and proteinase K. The antimicrobial component of the cell-free supernatant is proteinaceous in nature (Table 1).

### Identification and purification of bacteriocins from P. acidilactici

**Fig. 2:**
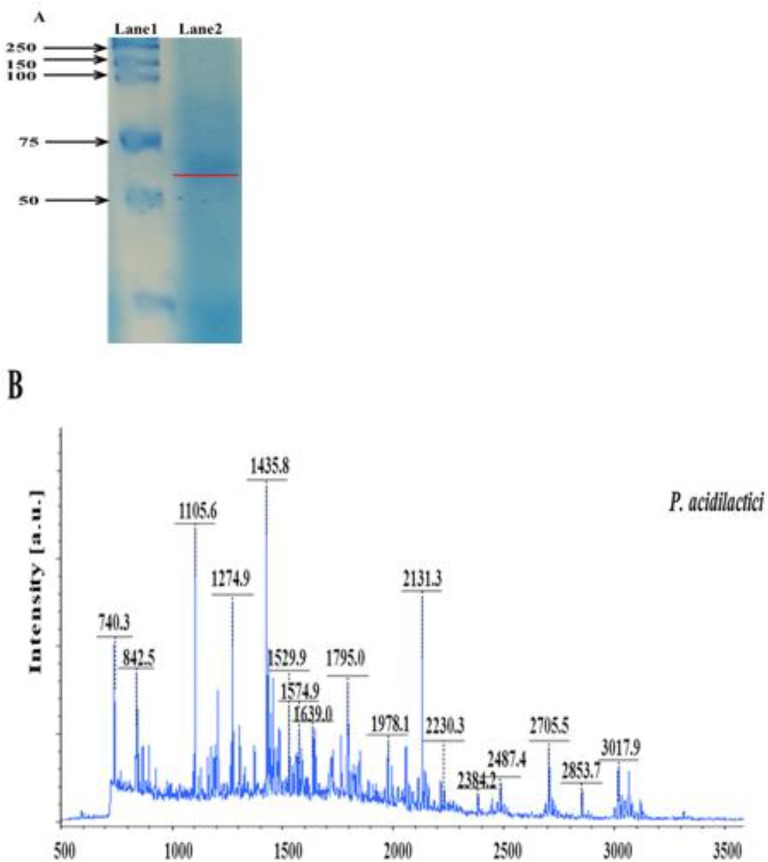
Identification of bacteriocin like substrate from *P. acidilactici*. (A) Molecular weight of the purified bacteriocin from *Pediococcus* strains by SDS-PAGE. (Lane 1: Protein marker Lane2: purified fraction from *P. acidilactici*); (B) MALDI TOF/MS spectral profiles of *Pediococcus* strains. Bacteriocin like substrate from *P. acidilactici* was identified as putative penicillin binding protein

The purified fractions from *P. acidilactici* were separated on a 12% SDS-PAGE gel. A single band was observed by Coomassie Brilliant blue G-250 staining (Fig.2).

The inhibitory activity of band was confirmed by ZOI analysis against *E.coli* (data not shown). The molecular mass of the excised gel from *P. acidilactici* was approximately 55–75 kDa. Of the 17 theoretical peptide masses obtained from mass spectrogram, 5 peptide masses on further analysis using MASCOT search revealed identity with penicillin binding protein (PBP) (score 29.4%) (Fig. 2B). This confirmed that the bacteriocin isolated from *P. acidilactici* was a putative PBP, with molecular weight of 51170 Da.

## Discussion

The main objective of this study was to isolate and characterize the bacteriocin-producing *Pediococcus* strains from Korean traditional nuruk. The isolated strains exhibited antimicrobial activities against different gram-positive and gram-negative pathogens, such as *K. pneumoniae, S. enterica, B. subtilis, B. cereus, E. coli*, and *S. flexneri*.

The phylogenetic analysis of the strains based on their 16S rRNA gene sequences is depicted in Fig. 1. Based on the homology analysis with GenBank reference strains, the isolated *Pediococcus* strains were identified as *P. acidilactici*, with close resemblance to the strains *P. acidilactici* NBRC 3885 (5). Isolation of *Pediococcus* strains has been reported from many fermented foods and dairy products. For instance, *P. acidilactici* Kp10 was isolated from milk product (17), *P. acidilactici* 13 from Turkish sucuk (18). The present study is the first report on the isolation of bacteriocins from *P. acidilactici* from the Korean traditional nuruk.

The bacteriocins isolated from *P. acidilactici* was sensitive to papain and trypsin treatments, confirming the proteinaceous nature of the component. Similar observations have been reported for other bacteriocins (2). PA-1 has been reported to be sensitive to proteinase K and trypsin treatments (15). Moreover, *P. acidilactici* SJ-1 was sensitive to pepsin (19). The results also reported the molecular mass of the purified bacteriocin in the range of 55–75 kDa, showing a strong resemblance to Class III bacteriocins, which are high molecular weight (>30 kDa), heat-labile proteins (19).

MALDI TOF/MS analysis revealed the presence of a putative penicillin binding protein as the major bacteriocin in *P. acidilactici*. Similar PBPs were identified from *Burkholderia cenocepacia* (20), *Acinetobacter baumannii* (21), *Enterococcus hirae* R40 (22), *Mycobacterium tuberculosis* (14, 23), and *E. coli* (24). Formation of PBP is catalyzed by the polymerization of the glycan strand (transglycosylation), followed by cross-linking between the glycan chains (transpeptidation). PBPs play a crucial role in the biosynthesis of peptidoglycans, which are essential components of the bacterial cell wall. Besides, PBPs also contribute to carbapenem resistance (16). The various bacteria like *E. coli* K-12, *Pseudomonas aeruginosa* and *Streptococcus pneumonia* were directly associated with alteration in PBP and β-lactam resistance (21). PBPs play a crucial role in the antibacterial activity against pathogenic bacteria. This is the first report on identification of such protein from a Korean traditional nuruk.

## Conclusion

The screening of a bacteriocin producing strains from Korean traditional nuruk led to isolation of novel active strain, identified as *P. acidilactici*. The bacteriocins exhibited broad host range activities against various gram-positive and gram-negative bacteria. The bacteriocin from *P. acidilactici* was identified as PBP. The strains have potential applications as preservatives in food industry. However, further experiments like utilization of the strains in fermentation processes or using the antimicrobial substance in the form of a bioformulation are required to explore the great potential of the strains.

## Ethical considerations

Ethical issues (Including plagiarism, informed consent, misconduct, data fabrication and/or falsification, double publication and/or submission, redundancy, etc.) have been completely observed by the authors.
